# Koala immunogenetics and chlamydial strain type are more directly involved in chlamydial disease progression in koalas from two south east Queensland koala populations than koala retrovirus subtypes

**DOI:** 10.1038/s41598-020-72050-2

**Published:** 2020-09-14

**Authors:** Amy Robbins, Jonathan Hanger, Martina Jelocnik, Bonnie L. Quigley, Peter Timms

**Affiliations:** 1 Genecology Research Centre, University of the Sunshine Coast, 90 Sippy Downs Drive, Sippy Downs, QLD 4556 Australia; 2 Endeavour Veterinary Ecology Pty Ltd, 1695 Pumicestone Road, Toorbul, QLD 4510 Australia

**Keywords:** Bacterial infection, Conservation biology

## Abstract

Chlamydial disease control is increasingly utilised as a management tool to stabilise declining koala populations, and yet we have a limited understanding of the factors that contribute to disease progression. To examine the impact of host and pathogen genetics, we selected two geographically separated south east Queensland koala populations, differentially affected by chlamydial disease, and analysed koala major histocompatibility complex (MHC) genes, circulating strains of *Chlamydia pecorum* and koala retrovirus (KoRV) subtypes in longitudinally sampled, well-defined clinical groups. We found that koala immunogenetics and chlamydial genotypes differed between the populations. Disease progression was associated with specific MHC alleles, and we identified two putative susceptibility (*DCb* 03, *DBb* 04) and protective (*DAb* 10, *UC* 01:01) variants. Chlamydial genotypes belonging to both Multi-Locus Sequence Typing sequence type (ST) 69 and *omp*A genotype F were associated with disease progression, whereas ST 281 was associated with the absence of disease. We also detected different *omp*A genotypes, but not different STs, when long-term infections were monitored over time. By comparison, KoRV profiles were not significantly associated with disease progression. These findings suggest that chlamydial genotypes vary in pathogenicity and that koala immunogenetics and chlamydial strains are more directly involved in disease progression than KoRV subtypes.

## Introduction

Chlamydial disease is a significant contributing factor affecting population viability in some declining northern Australian koala populations^1^. *Chlamydia pecorum* is endemic in almost all koala populations, however, in northern koala populations, it is highly prevalent and causes more severe disease^2^. In south east Queensland (SE Qld), a population prevalence for chlamydial infection and disease of up to 73% and 53%, respectively, has been reported^3,4^. Chlamydial infections predominantly cause inflammatory and fibrotic disease in the eyes and urogenital tract of susceptible koalas^5–7^, and can result in blindness, infertility and death^2^. Consequently, population management programs that incorporate chlamydial disease control, as well as manage concurrent threats, can reverse the extinction trajectory of these declining populations^1,8^. The success of these management interventions relies on a thorough understanding of chlamydial epidemiology in koalas, however this understanding has been limited by a lack of comprehensive, longitudinal population studies^9^ and the complexity of host–pathogen interactions.

Chlamydial disease progression in koalas is likely multifactorial, and associations with the chlamydial strain and infection load, co-infection with koala retrovirus (KoRV) and variation in koala immune genes have been identified as contributing factors. Chlamydial strain typing in one SE Qld population, using a fragment of the *omp*A gene, found that koalas with genotype F/F´ infections at their urogenital tract site and genotype E´ infections at their ocular site were significantly more likely to progress to disease^9^. Conversely, koalas with genotype G infections generally did not progress to disease^10^ and Victorian (Vic) koalas with genotype B infections only displayed mild signs of disease^11^. Further, chlamydial strain typing in a New South Wales (NSW) population, using Multi-Locus Sequence Typing (MLST), suggested that increased disease expression in the population may have been due to the introduction of a novel chlamydial strain^12^. Another identified predictor of disease progression in koalas is the load of chlamydial organisms in the urogenital tract^13^. A longitudinal study in SE Qld found a significant correlation between higher urogenital tract infection loads and progression to disease^9^. In addition, significantly higher infection loads were detected in the eyes and urogenital tract of hospitalised SE Qld koalas with active disease^14^. Co-infection with particular KoRV subtypes has also been linked to disease progression, with KoRV-B infection linked to chlamydial disease in SE Qld koalas^15^ and KoRV-A infection linked to ‘wet bottom’ in Vic koalas^16^. Finally, Class II major histocompatibility complex genes (MHC) have been associated with chlamydial disease in koalas, with the absence of *DBb* allele 03^13^ and the presence of *DAb* allele 10 and *DBb* allele 04^17^ linked to disease. The contrasting pattern of chlamydial disease prevalence and severity between northern and southern koala populations may be due to regional differentiation of these host and pathogen genotypes^4,18,19^.

Several suggested biogeographical barriers have been identified on the eastern coast of Australia, where climate-driven habitat fragmentation in the late Pleistocene era is thought to have contributed to the genetic divergence of some species^20–22^. One of these biogeographical barriers, the Brisbane Valley barrier (BVB), might have impacted the phylogeography of the koala^23^. Although a recent large-scale analysis of mitochondrial DNA control regions (CR) in koalas supported historical genetic connectivity across their range, it did identify four weakly differentiated lineages in SE Qld^24^. These occurred in three geographic clusters, with two northern lineages separated from a central lineage by the BVB^24^. These lineages were also supported by a study using single nucleotide polymorphisms (SNPs) mapped to the koala genome^23^. As ongoing habitat fragmentation continues to drive genetic divergence in these lineages, both the koala and its key infectious agents, *C. pecorum* and KoRV, are likely to be affected. This divergence provides a unique opportunity to study the impact of specific genetic variants on chlamydial disease outcomes.

To better understand the major factors associated with chlamydial disease progression in koalas, we longitudinally sampled two SE Qld koala populations known to be differentially affected by chlamydial disease and occurring approximately 70 kms apart on either side of the BVB^23,24^. Koalas from these populations were classified into well-defined clinical groups and their infecting chlamydial genotypes, immunogenetics and KoRV subtypes were characterised. Assessment of these three major parameters determined that koala immunogenetics and chlamydial genotype had the most direct effect on koalas progressing to chlamydial disease, in these populations. This improved understanding of chlamydial epidemiology will assist with koala population management, including translocation risk assessments, habitat restoration and selective breeding programs, maximising the effectiveness of management interventions and ensuring successful conservation outcomes.

## Results

In this study, koalas from two geographically separated populations in SE Qld, at the Moreton Bay site (MB)^9^ and the Old Hidden Vale site (HV), underwent regular field monitoring and clinical examinations approximately every 6 months (or more frequently if required for health or welfare concerns). Blood samples, ocular conjunctival swabs and a urogenital tract swab were collected during each clinical examination. From these samples, *C. pecorum* load and genotype, koala MHC immunogenetics and KoRV proviral subtypes were determined. These results were evaluated in the context of clinical records compiled at the time of sample collection, which included chlamydial disease status.

### Chlamydial epidemiology at each study site

#### The overall prevalence of chlamydial infection and disease differed between the study sites

Longitudinal monitoring of 24 HV koalas (over 113 individual sampling points) identified 24 chlamydial infections for strain typing analysis and eight new chlamydial infections for disease progression analysis. This complemented longitudinal monitoring of 148 MB koalas (over 479 individual sampling points)^9^ that identified 76 chlamydial infections for strain typing analysis and 38 new chlamydial infections for disease progression analysis. Overall, there was a significantly higher prevalence of infection at HV (58%, 14/24) compared to MB (35%, 89/254)^26^ (Fisher’s exact test *p* = 0.028) (Table 1), as well as a significantly higher prevalence of disease at HV (58%, 14/24) compared to MB (27%, 75/279) ^26^ (Fisher’s exact test *p* = 0.002).

**Table 1:** A comparison of chlamydial epidemiology between the Moreton Bay site (MB) and the Old Hidden Vale site (HV).

Parameter	Moreton Bay site (MB)	Old hidden vale site (HV)	Fisher's exact test significance
Prevalence of infection^#^	35% (89/254)*	58% (14/24)	***p = 0.028***
Prevalence of disease^#^	27% (75/279)*	58% (14/24)	***p = 0.002***
‘Severe’ outcomes^^^	10% (16/158)**	21% (3/14)	*p* = 0.189
‘Severe’ outcomes associated with prior chlamydial exposure^^^	Yes (5/5)**	No (0/3)	***p = 0.018***
‘Severe’ outcomes associated with cystitis^^^	Yes**	Yes	ND
Female reproductive tract disease^^^	63% (62/98)**	86% (6/7)	*p* = 0.417
Ongoing or repeated chlamydial exposure necessary for female reproductive tract disease^^^	No**	No	ND
Female reproductive tract disease with detectable chlamydial infection^^^	53% (16/30)**	100% (6/6)	*p* = 0.063
Long-term 'protection' against reinfection after successful antimicrobial treatment^^^	No**	No	ND
New infections detected	38**	8	ND
New infections that progressed to disease	66% (25/38)**	100% (8/8)	*p* = 0.084
New infections that were resolved	29% (11/38)**	0% (0/8)	*p* = 0.169
Urogenital tract site MLST sequence types	69, 202	69, 202, 281	ND
Ocular site MLST sequence types	202	69	ND
Urogenital tract site MLST sequence type 69 prevalence	12% (5/41)	59% (13/22)	***p < 0.001***
Urogenital tract site MLST sequence type 202 prevalence	88% (36/41)	14% (3/22)	***p < 0.001***
Urogenital tract site *omp*A genotypes	A´, E´, F, F´, G, E58**	F, E´	ND
Ocular site *omp*A genotypes	E´, G**	F, A´	ND
Urogenital tract site *omp*A genotype F prevalence	12% (6/50)**	82% (18/22)	***p < 0.001***
Urogenital tract site *omp*A genotype E´ prevalence	64% (32/50)**	18% (4/22)	***p < 0.001***
Different MLST sequence types detected over time in long-term infections	No (0/7)	No (0/3)	*p* = 1.000
Different *omp*A genotypes detected over time in long-term infections	Yes (3/7)**	No (0/3)	*p* = 0.528
Significantly higher urogenital tract infection loads when infection and disease were detected at the same time point	Yes**	Yes	ND
Significantly higher urogenital tract infection loads in recently acquired infections	Yes**	Yes	ND

Previously published in * Quigley et al.^26^ and ** Robbins et al.^9^, # based on the latest sample (during 2013–2014 for MB^26^ and during 2018–2019 for HV) for koalas tested more than once, ^ see Supplementary Information for more details, MLST denotes Multi-Locus Sequence Typing, E58 is an *omp*A sequence fragment that is identical to the bovine E58 strain, ND denotes not determined.

#### Chlamydial disease progression was common at both study sites

A total of eight HV koalas met our study inclusion criteria for disease progression analysis by having a new chlamydial infection detected at the ocular (n = 1) or urogenital tract site (n = 7) by quantitative polymerase chain reaction (qPCR) over a period of 18 months. These koalas had no evidence of chlamydial infection (infection loads below detection limit) or disease (clinical examination within normal limits) at that anatomical site at their previous clinical examination. If disease was detected at their first clinical examination, they were excluded from disease progression analyses only (unless it was their first sampling as an independent offspring, n = 1).

Interestingly, all of the new chlamydial infections at HV (100%, 8/8) progressed to disease, which was not significantly different to the number of new chlamydial infections at MB that progressed to disease (66%, 25/38)^9^ (Fisher’s exact test *p* = 0.084) (Supplementary Fig. S1). For six of these new chlamydial infections at HV (one ocular and five urogenital tract), the infection was detected at the same clinical examination as disease. For the other two new chlamydial infections at HV (both urogenital tract), the infection was present at a clinical examination 2.5 months and 4 months before disease was detected.

#### The urogenital tract infection load dynamics were similar at both study sites

The urogenital tract infection load (*C. pecorum* genome copies/µL) in both HV and MB^9^ koalas was significantly higher when infections were detected at the same clinical examination as disease (1,028,000 copies/µL, range 11,400–4,760,000 copies/µL), in comparison to infections that were present for one or more consecutive clinical examinations before disease was detected or infections that did not progress to disease (600 copies/µL, range 49–522,800 copies/µL) (Mann–Whitney U = 3, *p* = 0.030). Similarly, the urogenital tract infection load in both HV and MB^9^ koalas was significantly higher when koalas acquired a new chlamydial infection (within the last three months) (1,834,000 copies/µL, range 52,400–4,760,000 copies/µL), compared to koalas who had long-term infections (present for more than three months) (724 copies/µL, range 35–7,142 copies/µL) (Mann–Whitney U = 0, *p* = 0.010). Interestingly, the urogenital tract infection load was significantly higher at HV (1,028,000 copies/µL, range 11,400–4,760,000 copies/µL) compared to MB (3,824 copies/µL, range 138–1,340,000 copies/µL) when infections were detected at the same clinical examination as disease (Mann–Whitney U = 11, *p* = 0.003). In contrast, the urogenital tract infection load was not significantly different between the study sites (HV 600 copies/µL, range 49–522,800 copies/µL vs MB 794 copies/µL, range 16–13,900 copies/µL) when infections were present for one or more consecutive clinical examinations before disease was detected or infections did not progress to disease (Mann–Whitney U = 28, *p* = 0.703).

#### The prevalence of chlamydial strains, as determined by Multi-Locus Sequence Typing, differed between the study sites

**Figure 1:**
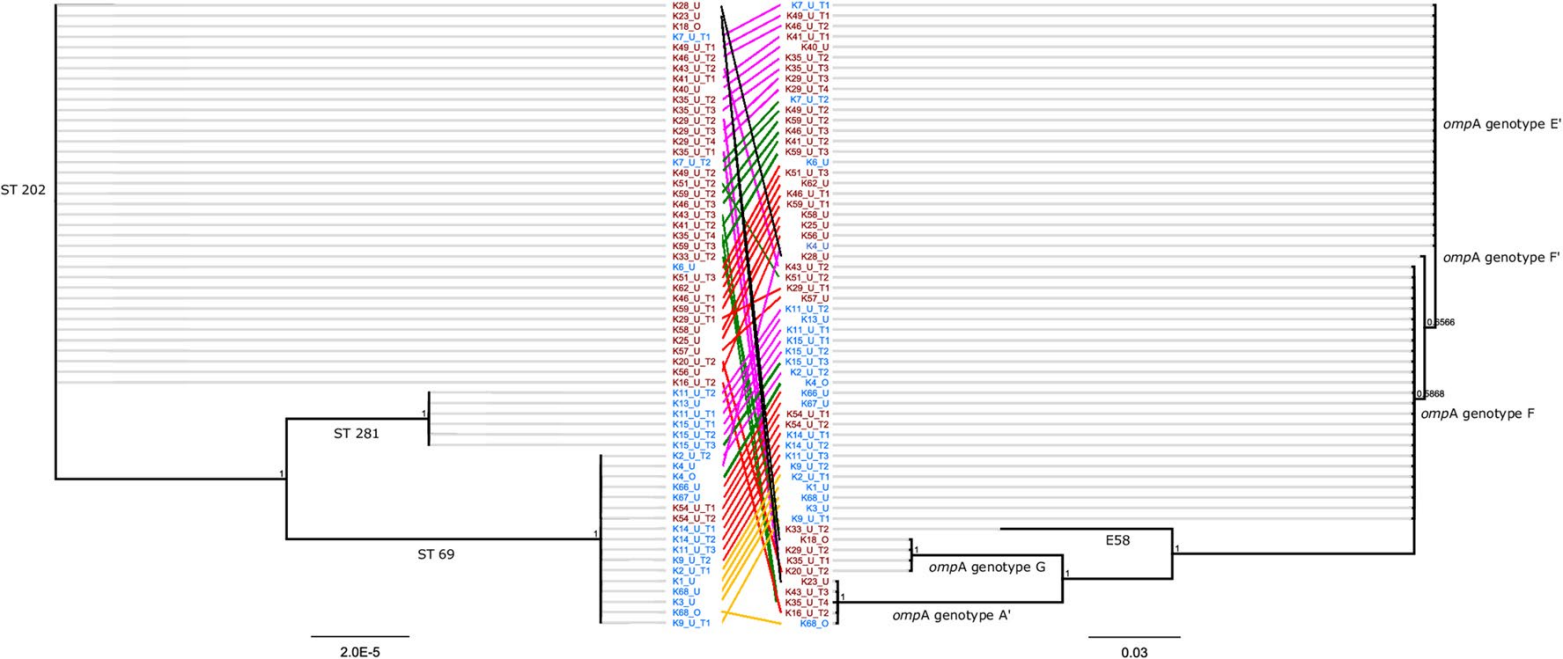
Mid-point rooted Bayesian phylogenetic trees constructed using the concatenated Multi-Locus Sequence Typing alignment (ST) (left) and *omp*A alignment (right) detected at the Moreton Bay site (MB—in red) and the Old Hidden Vale site (HV—in blue) (K# denotes koala number, U or O for urogenital vs ocular sample, T# for timepoint from longitudinal samples, clinical groups are marked with coloured lines; black denotes the resolver group, pink denotes the chronic infection group, green denotes the diseased after chronic infection group, red denotes the infected and diseased group, yellow denotes the diseased at first exam group), E58 is an *omp*A sequence fragment that is identical to the bovine E58 strain (created using Inkscape 0.92.3, https://inkscape.org/).

Overall, 69 *C. pecorum*-positive samples, comprised of 45 samples from MB (4 ocular and 41 urogenital tract samples from 25 koalas) and 24 samples from HV (2 ocular and 22 urogenital tract samples from 14 koalas), were analysed using a *C. pecorum*-specific MLST scheme^27^. Three sequence types (STs) were detected in this study: ST 69, ST 202 and a novel ST (ST 281). ST 69 and ST 202 were detected at both study sites, however their prevalence at each study site was significantly different (Fig. 1). ST 69 was the most prevalent ST at HV, detected in 63% of total samples (15/24) and in 59% of urogenital tract site samples (13/22). ST 69 was significantly less prevalent at MB, detected in 11% of total samples (5/45) and in 12% of urogenital tract site samples (5/41) (overall and urogenital tract site Fisher’s exact test *p* < 0.001). In contrast, ST 202 was the most prevalent ST at MB, detected in 87% of total samples (39/45) and in 88% of urogenital tract site samples (36/41). ST 202 was significantly less prevalent at HV, detected in 13% of total samples (3/24) and in 14% of urogenital tract site samples (3/22) (overall and urogenital tract site Fisher’s exact test *p* < 0.001). The novel ST at HV, ST 281, was detected at the urogenital tract site in three koalas, representing 25% of total samples (6/24) and 27% of urogenital tract site samples (6/22). In comparison to the other previously described koala *C. pecorum* STs, ST 202 clustered in a well-supported, closely related clade with STs commonly detected in Qld and NSW koalas, including ST 220, ST 199 and ST 70 (Supplementary Fig. S2). ST 69 and ST 281, however, clustered into their own clades.

#### The prevalence and diversity of chlamydial strains, as determined by *omp*A genotyping, differed between the study sites

To compare *ompA* genotypes between MB and HV, all 24 *C. pecorum*-positive HV samples were genotyped and compared to previously reported MB genotypes (62 total samples, 12 ocular and 50 urogenital tract samples)^9^ (Fig. 1). The *omp*A genotype F was the most prevalent *omp*A genotype at HV, detected in 79% of total samples (19/24) and in 82% of urogenital tract site samples (18/22). The *omp*A genotype F was significantly less prevalent at MB, detected in 10% of total samples (6/62) and in 12% of urogenital tract site samples (6/50)^9^ (overall and urogenital tract site Fisher’s exact test *p* < 0.001). In contrast, the *omp*A genotype E´ was the most prevalent *omp*A genotype at MB, detected in 60% of total samples (37/62) and in 64% of urogenital tract site samples (32/50)^9^. The *omp*A genotype E´ was significantly less prevalent at HV and was the second most prevalent *omp*A genotype at this study site, detected in 17% of total samples (4/24) and in 18% of urogenital tract site samples (4/22) (overall and urogenital tract site Fisher’s exact test *p* < 0.001). The only other *omp*A genotype detected at HV was the *omp*A genotype A´, which was detected in a single ocular site sample.

Although a diverse range of *omp*A genotypes was detected at the urogenital tract site at MB, including A´, E´, F, F´, G and an *omp*A sequence fragment identical to that of the bovine E58 strain^9^, only *omp*A genotypes F and E´ were detected at the urogenital tract site at HV (Supplementary Fig. S2). For multifocal infections (ocular and urogenital tract sites) at HV (n = 2), the *omp*A genotype detected at each anatomical site differed. In one case, *omp*A genotype A´ was detected at the ocular site and *omp*A genotype F was detected at the urogenital tract site, and in the other, *omp*A genotype F was detected at the ocular site and *omp*A genotype E´ was detected at the urogenital tract site. Overall, the diversity in *omp*A, where six genotypes were characterised, was higher than the diversity in STs, where only three STs were characterised (Fig. 1). The *C. pecorum* plasmid was detected in all chlamydial strains except for a single ocular site sample from one koala at MB (see Supplementary Information for more details).

#### The dynamics of chlamydial strains during long-term infections differed between the strain typing methods

**Figure 2:**
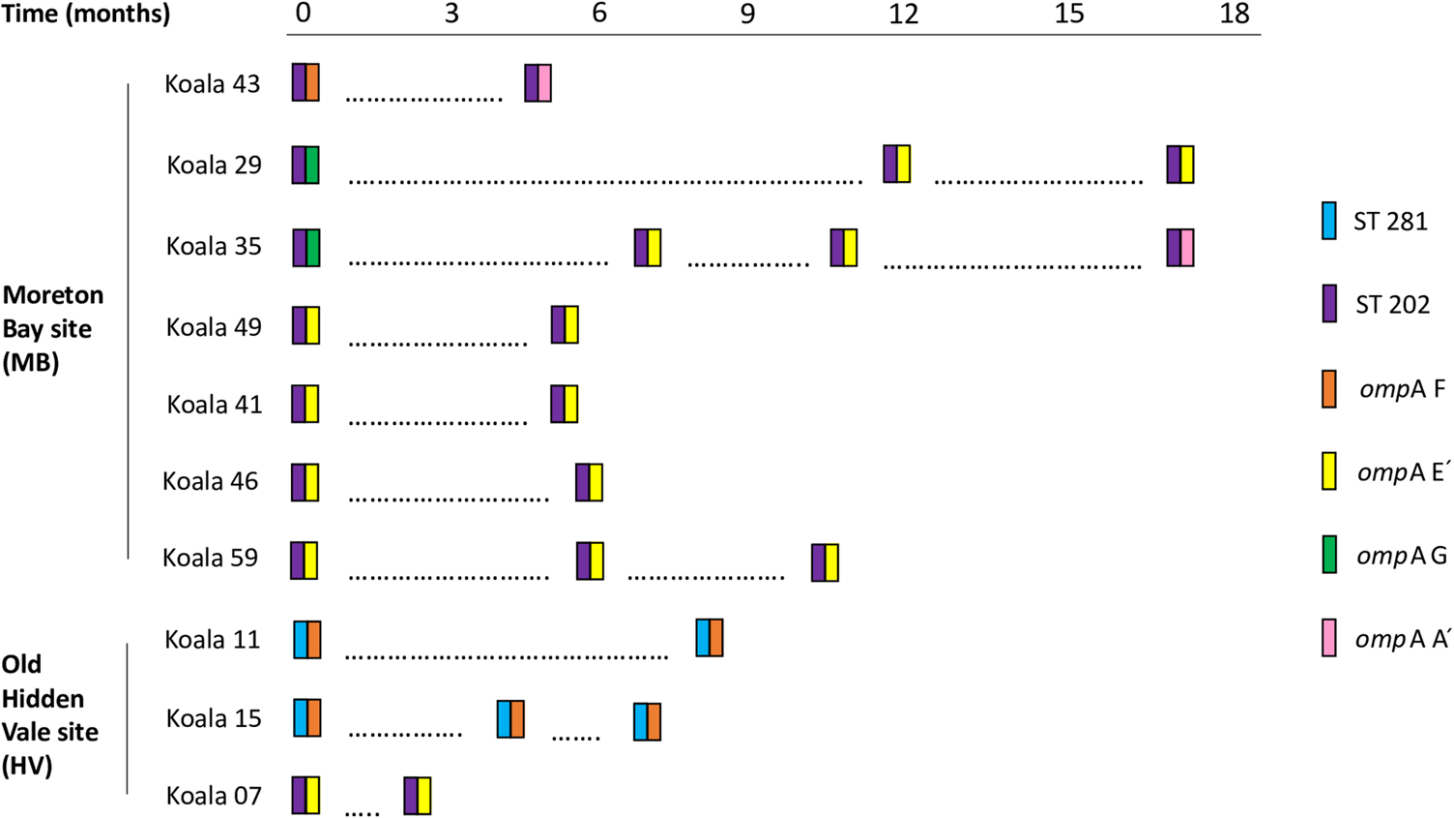
Schematic of long-term infections over time, demonstrating changing *omp*A genotypes but identical Multi-Locus Sequence Typing sequence types (STs) (boxes denote sampling points and the time between sampling points is indicated in the top scale bar) (created using Inkscape 0.92.3, https://inkscape.org/).

A total of 10 long-term urogenital tract infections were analysed with both strain typing methods, three at HV and seven at MB (Fig. 2). Although identical STs were detected over time in all long-term urogenital tract infections (100%, 10/10), genetically distinct *omp*A genotypes were detected over time in three of these long-term urogenital tract infections (30%, 3/10) at MB^9^. ST 202 was always detected in these infections, however the *omp*A genotype changed from F to A´ (4.5 months later) in one case, G to E´ (12 months later) in another case and G to E´ (7 months later) and again to A´ (10.5 months later) in the last case. An additional six *omp*A sequences from ocular (n = 1) and urogenital tract site (n = 5) samples collected from MB koalas, not previously reported by us^9^, were included in this analysis. Unfortunately, although the *omp*A fragment was amplified in a further two ocular and six urogenital tract site samples, we were not able to resolve the *omp*A sequences from these chromatograms.

### Impact of chlamydial genotype on disease progression

#### Urogenital tract disease progression at each study site was associated with the chlamydial strain

At MB, ST 69 was significantly more prevalent in koalas that resolved their infections without progressing to disease (60%, 3/5) (the *resolver* group) compared to koalas that did not resolve their infections (6%, 2/36) (the *chronic infection*, the *diseased after chronic infection* and the *infected and diseased* groups combined) (Fisher’s exact test *p* = 0.009). Interestingly, at HV, ST 281 was significantly more prevalent in koalas that had not progressed to disease (56%, 5/9) (the *chronic infection* group) compared to koalas that had progressed to disease (1/12, 8%) (the *infected and diseased* and the *diseased after chronic infection* groups combined) (Fisher’s exact test *p* = 0.046). Further, when STs and *omp*A genotypes were concatenated to form a Multi-Locus Sequence Analysis (MLSA) type, MLSA type 2 (ST 281 with *omp*A genotype F) was significantly more prevalent in koalas that had not progressed to disease (56%, 5/9) compared to koalas that had progressed to disease (8%, 1/12) (Fisher’s exact test *p* = 0.046). In contrast, MLSA type 1 (ST 69 with *omp*A genotype F) was significantly more prevalent in koalas that had progressed to disease (75%, 9/12) compared to koalas that had not progressed to disease (22%, 2/9) (Fisher’s exact test *p* = 0.030).

When urogenital tract disease progression and chlamydial strains were analysed across both study sites combined (41 MB and 21 HV koalas for STs, 35 MB and 21 HV koalas for *omp*A genotypes and MLSA types), there were no significant differences in the prevalence of STs, *omp*A genotypes or MLSA types in koalas that had progressed to disease compared to koalas that had not progressed to disease.

### Immunogenetic profiles at each study site

#### The major histocompatibility complex haplotypes were genetically diverse between the study sites

To investigate MHC gene diversity at each site, 60 koalas were selected from MB (30 koalas from our previous chlamydial epidemiology analyses (see Robbins et al.^9^ for more details) and 30 additional healthy koalas) and 20 koalas were selected from HV (sexually mature koalas undergoing monitoring). The MHC allele diversity in two Class I genes (*UA* and *UC*) and four Class II genes (*DAb*, *DBb*, *DMb* and *DCb*) was determined (Supplementary Fig. S3).

**Figure 3:**
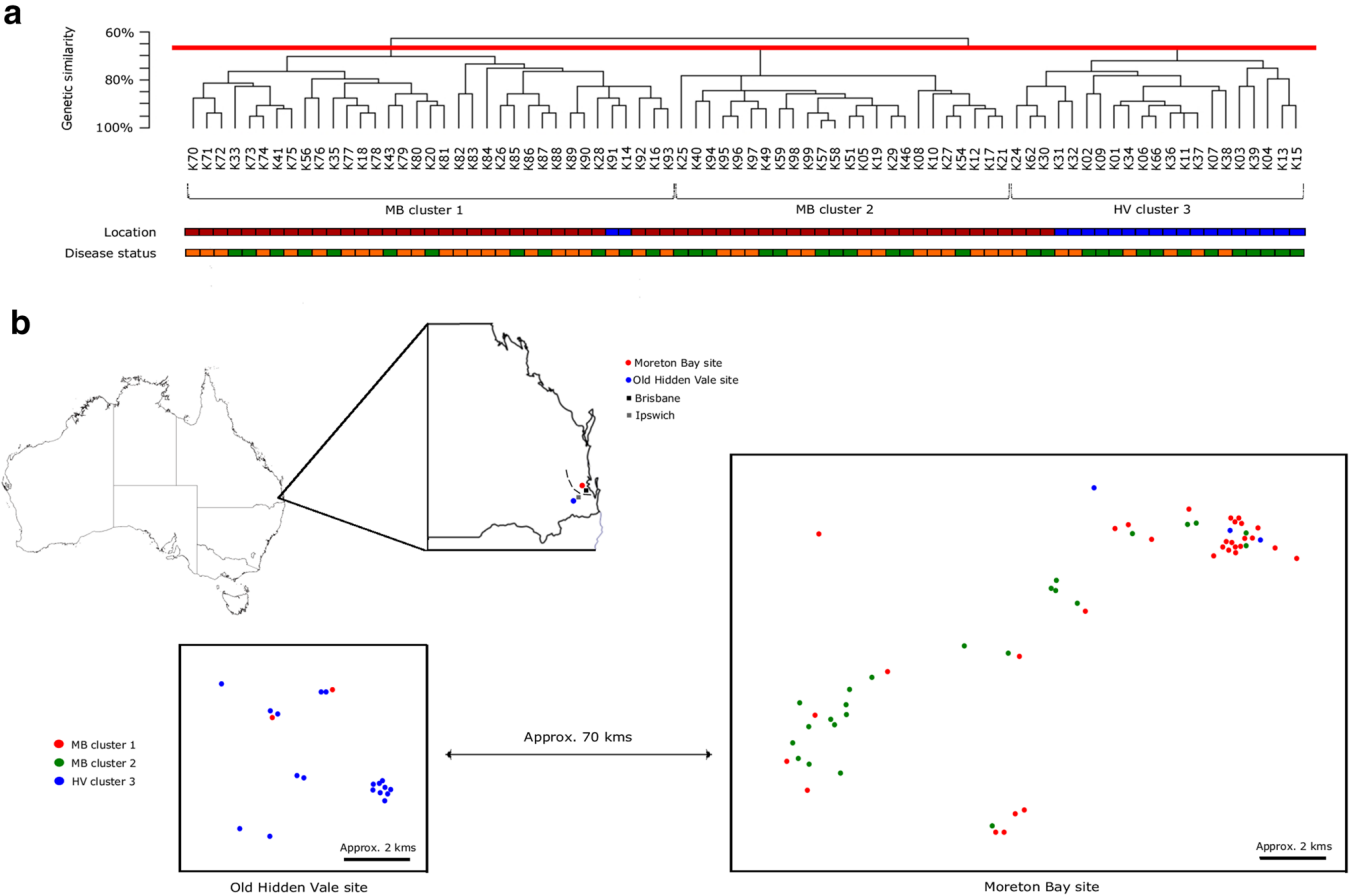
(**a**) Cluster dendrogram of major histocompatibility complex (MHC) haplotypes indicating clustering by study site but not by disease progression (red line denotes > 65% genetically identical, red squares denote Moreton Bay site (MB), blue squares denote Old Hidden Vale site (HV), green squares denote developed disease during monitoring, orange squares denote did not develop disease during monitoring) (**b**) Schematic showing study sites and capture location of koalas within MHC haplotype clusters (dashed line denotes the Brisbane Valley biogeographical barrier) (created using Inkscape 0.92.3, https://inkscape.org/).

Overall, MHC haplotypes clustered based on study site (Fig. 3). Koalas grouped into three MHC haplotype clusters based on more than 65% genetic similarity. Two of these clusters represented MB koalas while the third cluster represented HV koalas. There was a tendency for MB koalas to belong to the same MHC cluster when they were captured from a similar geographical location within the 13 km site, suggesting some geographical sub-population structure. MB clusters 1 and 2 overlapped, however, and there are records of both natural dispersal and koala translocations within this study site. Interestingly, despite the separation of the MB and HV koalas by the BVB^23,24^ (and approximately 70 kms), three MB koalas genetically grouped within HV cluster 3 while two HV koalas genetically grouped within MB cluster 1.

#### The prevalence of major histocompatibility complex alleles differed between the study sites

Overall, 61 MHC alleles were detected in this study (Supplementary Fig. S3). In total, 21 MHC alleles were only detected at MB, 14 MHC alleles were only detected at HV and 26 MHC alleles were detected at both study sites. There were eight MHC alleles that had a significantly higher prevalence at each study site (Fisher’s exact test *p* < 0.05), with *UA* 10:01, *UA* 14:01, *UC* 01:01, *UC* 05:02, *DAb* 10, *DAb* 19, *DBb* 03 and *DMb* 04 significantly more prevalent in MB koalas and *UA* 08:01, *UA* 11:01, *UA* 17:01, *UC* 01:03, *DAb* 22, *DAb* 23, *DAb* 37 and *DBb* 04 significantly more prevalent in HV koalas (Supplementary Table S1). In addition, this study expanded our knowledge of koala MHC allele diversity, with a total of 28 previously unreported MHC alleles being identified (15 at MB, 12 at HV and one at both study sites) (Supplementary Fig. S3).

### Impact of host genetics on disease progression

#### Urogenital tract disease progression was associated with major histocompatibility complex alleles

At each study site, the prevalence of only one individual MHC allele was significantly different between koalas that progressed to disease and koalas that did not progress to disease. At MB, the Class II allele *DAb* 10 was significantly more prevalent in koalas that did not progress to disease (50%, 17/34) compared to koalas that progressed to disease (13%, 3/23) (Fisher’s exact test *p* = 0.005). In contrast, at HV, the Class II allele *DCb* 03 was significantly more prevalent in koalas that progressed to disease (75%, 9/12) compared to koalas that did not progress to disease (14%, 1/7) (Fisher’s exact test *p* = 0.020).

When urogenital tract disease progression and koala immunogenetics were analysed across both study sites combined (57 MB and 19 HV koalas), the prevalence of four individual MHC alleles was significantly different between koalas that progressed to disease and koalas that did not progress to disease. The Class II allele *DCb* 03 was significantly more prevalent in koalas that progressed to disease (66%, 23/35) compared to koalas that did not progress to disease (34%, 14/41) (Fisher’s exact test *p* = 0.011), and koalas with *DCb* allele 03 were 3.70 times (95% CI 1.43–9.56 times) more likely to progress to disease. The Class II allele *DBb* 04 was also significantly more prevalent in koalas that progressed to disease (11%, 4/35) compared to koalas that did not progress to disease (0%, 0/41) (Fisher’s exact test *p* = 0.041). In contrast, the Class II allele *DAb* 10 was significantly more prevalent in koalas that did not progress to disease (42%, 17/41) compared to koalas that progressed to disease (11%, 4/35) (Fisher’s exact test *p* = 0.004), and koalas with *DAb* allele 10 were 5.49 times (95% CI 1.63–18.46 times) less likely to progress to disease. Finally, the Class I allele *UC* 01:01 was also significantly more prevalent in koalas that did not progress to disease (100%, 41/41) compared to koalas that progressed to disease (89%, 31/35) (Fisher’s exact test *p* = 0.041).

MHC haplotypes were not, however, associated with overall disease progression, with koalas that progressed to disease at any anatomical site at any time during the period of monitoring distributed evenly throughout the genetic clusters (Fig. 3).

### Koala retrovirus profiles at each study site

#### The prevalence of most koala retrovirus subtypes, except KoRV-F, was similar between the study sites

To investigate the KoRV profiles of koalas at each study site, the KoRV proviral *env* gene was amplified and sequenced for the same 80 koalas used for the MHC gene diversity analysis (60 MB koalas over 68 individual sampling points and 20 HV koalas over 22 individual sampling points). Overall, five KoRV subtypes were detected, with KoRV subtypes -A, -B, -D and -F detected at both study sites and KoRV-G only detected at MB.

When only the first time-point was included for koalas with longitudinal samples, KoRV-F was significantly more prevalent in MB koalas (77%, 46/60) compared to HV koalas (25%, 5/20) (Fisher’s exact test, *p* < 0.001) (Supplementary Fig. S4. There were no significant differences in the prevalence of any other KoRV subtype between the study sites. As expected, KoRV-A prevalence was 100% in both MB koalas (60/60) and HV koalas (20/20). The prevalence of KoRV-B was 40% (24/60) in MB koalas compared to 55% (11/20) in HV koalas (Fisher’s exact test *p* = 0.301), the prevalence of KoRV-D was 97% (58/60) in MB koalas compared to 100% (20/20) in HV koalas (Fisher’s exact test *p* = 1.000), and the prevalence of KoRV-G was 2% (1/60) in MB koalas compared to 0% (0/20) in HV koalas (Fisher’s exact test *p* = 1.000).

### Impact of co-infection with koala retrovirus on disease progression

#### Urogenital tract disease progression was not associated with koala retrovirus profiles

There were no significant differences in the prevalence, proportional abundance or diversity (number of OTUs per koala) of any KoRV subtype between koalas that had progressed to disease (the *infected and diseased* and the *diseased after chronic infection* groups combined) and koalas that had not progressed to disease (the *chronic infection* group) at either study site (35 MB and 13 HV koalas), or when urogenital tract disease progression and KoRV profiles were analysed across both study sites combined.

## Discussion

*C. pecorum* is highly prevalent in northern Australian koala populations and causes infertility and mortality in susceptible koalas, threatening the viability of these increasingly fragmented and declining populations^2^. Population management programs that incorporate chlamydial disease control may help to stabilise these populations^1^, however limitations in our understanding of chlamydial epidemiology might affect program efficiency and success. To address these limitations and build on our previous findings^9^, we analysed two unique, longitudinally-studied koala populations with well-defined clinical groups. These koala populations were geographically separated and differentially affected by chlamydial disease, allowing us to investigate several factors thought to contribute to chlamydial epidemiology. We found that in these two SE Qld koala populations, koala immunogenetics and chlamydial genotypes had a more direct impact on urogenital tract disease progression than KoRV proviral subtypes. Four individual MHC alleles were linked with overall urogenital tract disease progression, with the Class II alleles *DCb* 03 and *DBb* 04 significantly associated with progression to disease and the Class II alleles *DAb* 10 and Class I allele *UC* 01:01 significantly associated with remaining healthy. We also found that chlamydial strains appeared to vary in pathogenicity, and chlamydial genotypes belonging to both ST 69 and *omp*A genotype F were associated with urogenital tract disease progression whereas ST 281 was associated with the absence of urogenital tract disease progression. Finally, we also detected different *omp*A genotypes, but not different STs, over time, in long-term infections.

Although a complex range of factors is known to influence chlamydial disease progression in koalas, the koala’s immune response to *C. pecorum* plays a key role and the MHC loci are known to be important in this response^17^. Our analysis of MHC alleles and urogenital tract disease progression in SE Qld populations identified two putative susceptibility (*DCb* 03, *DBb* 04) and protective variants (*DAb* 10, *UC* 01:01). The Class II allele *DCb* 03 was significantly more prevalent in HV koalas that progressed to disease, and overall, disease progression was 3.70 times more likely in SE Qld koalas with the *DCb* allele 03. This finding contrasts with a modelling study in SE Qld that suggested that the absence of *DCb* allele 03 was associated with disease progression^13^, however disease progression at both the ocular and urogenital tract sites was combined in this analysis, and this may have impacted the findings. Further, overall, disease progression in SE Qld koalas was significantly associated with the Class II allele *DBb* 04. Interestingly, a study in NSW koalas reported significantly higher chlamydial-hsp60 antibody titres in koalas with *DBb* allele 04, suggesting this variant recognises and binds to c-hsp60 epitopes^17^. High c-hsp60 antibody titres in koalas have been associated with fibrous occlusion of the uterus or uterine tube, however their role in chlamydial pathogenesis in koalas remains unclear as they were also associated with lower levels of active inflammation and fewer chlamydial inclusions^28^. Human studies suggest that although the Class II MHC defined anti-chlamydial antibody response appears to be important for chlamydial clearance and c-hsp60 antibodies are protective, it does not always prevent pathology^29,30^.

In contrast, the Class II allele *DAb* 10 was significantly more prevalent in MB koalas that did not progress to disease, and overall, disease progression was 5.49 times less likely in koalas with *DAb* 10. In a study in NSW koalas, *DAb* allele 10 was associated with chlamydial infection and persistence^17^, however chlamydial infection only and diseased koalas were combined in this hospital data set analysis, and these factors might contribute to our contrary results. Overall, the Class I allele *UC* 01:01 was also significantly associated with the absence of disease progression in SE Qld koalas. The *UC* gene has been identified as a classical MHC gene in koalas and appears to be under diversifying selection, suggesting it might be important in the anti-chlamydial immune response^31^. Analysis of the koala genome showed that *UC* and *UE* genes were located only approximately 72 bp apart in the core MHC region in the genome and shared 95.5% similarity, likely originating as a gene duplication event^31^. Further, although limited variability in the *UE* gene was reported, it had tissue-specific expression in the testes (and thymus)^31^, indicating it is active at a site of chlamydial exposure. Taken together, these findings suggest that the *UE* gene should be included in subsequent investigations into associations between MHC gene polymorphisms and chlamydial disease progression. Finally, the significantly higher prevalence of putative protective variants (*DAb* 10 and *UC* 01:01) at MB and a putative susceptibility variant (*DBb* 04) at HV may have contributed to the significantly higher overall prevalence of chlamydial infection and disease at HV. Focused future study of the chlamydial epitopes that bind to these putative susceptibility and protective variants, as well as the cytokine profile and MHC gene expression levels in variant positive and negative koalas is necessary to better understand the role of koala immunogenetics in chlamydial disease progression.

Our findings also strengthen a growing body of data that support the infecting chlamydial strain as an important contributor for chlamydial disease progression in koalas^9,11,12,32^. Our approach was to combine a MLST scheme, which has been shown to be congruent with whole genome phylogeny and is a robust method for investigating genetic diversity^33^, with *omp*A genotyping, which appears to capture more genetic diversity due to the polymorphic nature of the *omp*A gene. This combined approach allows for fine-scale *C. pecorum* epidemiological investigations^12,34^. At this fine-scale, we determined that chlamydial genotypes belonging to both ST 69 and *omp*A genotype F appeared to be more pathogenic at HV and were associated with urogenital tract disease progression. Further, the significantly higher prevalence of this chlamydial strain at HV correlated with a higher overall prevalence of infection and disease in this population. This finding strengthens the known association of *omp*A genotype F with higher urogenital tract infection loads and chlamydial disease progression in koalas^9,11^. In contrast, ST 69 was associated with resolved urogenital tract infections at MB in this study, while ST 69 has previously been associated with clinical disease in NSW koalas^12^ as well as being detected in koalas without clinical signs of disease^33^. We also found that ST 281 appeared to be less pathogenic at HV and was associated with the absence of disease progression and milder urogenital tract disease once progression had occurred. The highly conserved housekeeping genes analysed for MLST are unlikely to directly be involved in pathogenicity, and whole genome sequencing (WGS) might be necessary to determine whether there are associations between particular STs and other potential virulence-associated loci, such as ORF 663 and *inc*A^35,36^, that are driving chlamydial epidemiology in koalas. In addition, the impact of these chlamydial genotypes on pathogenicity could be further investigated with in vitro studies.

The polymorphic *omp*A gene, encoding the surface-exposed major outer membrane protein, may undergo more rapid evolution than the rest of the chlamydial genome, due to selection pressure from the host immune system^37^. This feature can make the *omp*A gene very useful in longitudinal and multifocal infection analyses. We detected genetically distinct *omp*A genotypes over time in 30% of the long-term urogenital tract infections examined, and these were all at MB where we detected a variety of genetically diverse *omp*A genotypes. Further, genetically distinct *omp*A genotypes were detected in the eyes and urogenital tract of koalas at both study sites during multifocal infections. This compared to detected STs, which were identical both over time in long-term urogenital tract infections and at each anatomical site during multifocal infections and were less genetically diverse overall in comparison to *omp*A genotypes. Interestingly, although we were able to resolve all the *omp*A genotypes from our HV samples, regardless of infection load, we were not able to resolve the *omp*A genotypes from eight MB samples despite MLST being successful. Taken together, these findings could be consistent with mixed genotype infections^38^ and extensive recombination in the *omp*A gene^39,40^ driving chlamydial strain microevolution in koalas. WGS could address the limitations of amplicon sequencing for detecting mixed genotype infections^38^, allowing us to better characterise circulating chlamydial strain diversity and its impact on chlamydial epidemiology.

Our understanding of the relationship between koalas and KoRV is rapidly evolving as a growing number of koalas are evaluated with increasingly sensitive methods. KoRV was originally thought to be spreading southwards after it was introduced to northern koala populations less than 200 years ago^41^, but recent analyses suggest it crossed into koalas up to 49,000 years ago^42^ and a range of subtypes have been detected in both northern and southern koala populations^43^. Consistent with these analyses, we found a strikingly similar prevalence of KoRV subtypes at each of our study sites, suggesting that the spread of KoRV in this region occurred before gene flow between our populations was limited by the BVB^24^ and the rapid and widespread declines in koala populations 30,000–40,000 years ago^23^. Utilising deep sequencing of the KoRV proviral *env* gene and our well-defined clinical groups, we failed to detect any association between the proviral detection of any KoRV subtype, including KoRV-B^15^, and disease progression^16,44,45^. This suggests that the presence of KoRV provirus alone is not sufficient to influence chlamydial disease progression and that subtype expression might be more important^46^. Acknowledging that KoRV detection methods have different targets and sensitivities^43,46^, further study is clearly needed to tease out the complex interactions between this putative pathogen and its host.

Although our data were collected from longitudinal studies of two koala populations using identical protocols, several factors differed between the study sites that may have impacted our findings. Variation in environmental factors exists between MB and HV in terms of rainfall, soil, vegetation communities, sympatry with livestock and proximity to human habitation. While evidence linking ‘habitat quality’ and chlamydial disease progression is inconclusive^18,32^, climate stress can impact population health^47,48^ and HV was drought-affected at the time of our study. Many of the koalas at HV also shared their habitat with cattle, and the likelihood and frequency of cross-host transmission of chlamydial strains from livestock to koalas is currently unknown^33^. Hence, sympatric sampling is a priority for future research at this site. It is interesting to note that in our study, proximity to human habitation was not associated with a higher prevalence of chlamydial infection or disease^13^, and there was a significantly higher prevalence of chlamydial infection and disease at the rural HV compared to the peri-urban/urban MB. Finally, in contrast to MB where an estimated 95% of the resident koala population was monitored^4^, a much smaller proportion of koalas at HV was able to be recruited into the monitoring program and the sample size was limited.

In conclusion, our research of longitudinally studied koalas with well-defined clinical outcomes has shown that MHC genetics and chlamydial genotypes were more directly linked to chlamydial disease progression than subtype-specific KoRV proviral profiles. These findings provide new focus for investigations into susceptibility and protective immune phenotypes, informing selective breeding programs, and highlight the risks of poorly planned translocations and the restoration of habitat connectivity to naïve koalas. Overall, our research has also shown that chlamydial disease progression commonly occurs in SE Qld koalas, suggesting that chlamydial disease control should be incorporated into population management programs in this region if the threat of chlamydial disease is to be abated.

## Methods

### Study sites

This study utilised two monitored koala populations in SE Qld. North of Brisbane (27.0946° S, 152.9206° E), MB is peri-urban/urban koala habitat composed of open and closed forest and woodland that was undergoing development as part of a large-scale infrastructure project (see Hanger et al.^5^ for more details). West of Ipswich (27.6594° S, 152.4672° E), HV is rural koala habitat composed of open forest and grassland that included farmland and a nature reserve. These study sites are separated by approximately 70 kms and the BVB^23,24^ (Fig. 1).

### Animals

The koala populations in our study were part of population management programs conducted by Endeavour Veterinary Ecology Pty Ltd (see Hanger et al.^5^ for more details). Koalas underwent regular capture, telemetric monitoring and comprehensive clinical examinations under anaesthesia (see below), and were treated for chlamydial disease when detected (see Robbins et al.^49^ for more details). During clinical examinations, swab samples were collected from the ocular conjunctiva and urogenital sinus (females) or urethra (males) and blood samples were collected from the cephalic vein. All samples were stored at −20 °C before being transported to the lab for processing. Detailed field observations and clinical examination records were compiled for each koala during their period of monitoring, which occurred between 2013 and 2017 at MB and 2018 and 2019 at HV.

In total, 24 koalas were recruited from HV, which was 100% of the sexually mature koalas monitored at this site (Table 2). We also utilised data from 47 koalas in our previous study at MB^9^ and recruited an additional 30 healthy, chlamydial infection- and disease-free koalas at MB to serve as a control group for our MHC and KoRV analyses. Control koalas were matched for age, sex, location and reproductive status, although this was not always possible at HV due to limitations in the number of koalas available. Multiple time-points were available for some koalas with chlamydial infections, and these were utilised to investigate how chlamydial load and genotype and KoRV profiles changed over time.

**Table 2:** Clinical groups used for chlamydial genotyping, koala immunogenetics and koala retrovirus (KoRV) subtyping analyses at the Moreton Bay (MB) and Old Hidden Vale (HV) sites.

Analysis	Chlamydial genotyping		Koala immunogenetics and KoRV subtyping	
**Clinical group**	**Moreton Bay site (MB)**	**Old Hidden Vale site (HV)**	**Moreton Bay site (MB)**	**Old Hidden Vale site (HV)**
Healthy control koalas	NA	NA	30	7
Resolved infections	9	0	10	0
Chronic infections	13	8	11	4
Diseased after chronic infection	9	3	4	3
Infected and diseased	14	7	13	3
Diseased at first exam	0	6	0	5
**Total number of samples**	45	24	68	22

*NA* denotes not applicable.

### Assessing chlamydial disease progression

Comprehensive clinical examinations were performed on koalas under anaesthesia by experienced koala veterinarians, and included a thorough physical examination, sonographic examination of the urogenital tract (including kidneys), and cytological examination of blood, bone marrow, peritoneal fluid and urine sediment. Koalas were defined as progressing to chlamydial disease if signs of chlamydial disease were detected during a clinical examination and the koala had been diagnosed as healthy (chlamydial disease-free) at their previous clinical examination. The minimum requirement for the diagnosis for ocular chlamydial disease was conjunctivitis, however keratoconjunctivitis, corneal ulceration, neovascularisation, scleral injection, pannus formation and blepharitis were also observed. The minimum requirement for the diagnosis of cystitis was an inflammatory urine sediment, with or without thickening of the bladder wall sonographically (> 2 mm mean wall diameter), however a urine stained, damp rump, secondary dermatitis and decubital ulcers were also observed. The minimum requirement for the diagnosis of reproductive disease was sonographic changes in the reproductive tract. In females, this included pyometra or cystic dilatation in the reproductive tract and in males, this included orchitis or prostatic lesions.

### Sample analysis for *C. pecorum*

Ocular conjunctiva and urogenital tract swab samples were mixed with 500 µL of phosphate-buffered saline. Total DNA was extracted from a 200 µL aliquot of this swab suspension using a QIAamp DNA mini kit (Qiagen), according to the manufacturer’s instructions. The extracted DNA was then used to screen for *C. pecorum* DNA using a *C. pecorum*-specific qPCR assay that targets a 209 bp region of the conserved gene *CpecG_0573*^9,50^, and chlamydial plasmid DNA using a CDS5-specific qPCR assay that targets a 233 bp fragment of the *C. pecorum* plasmid (CDS5 or Pgp3 locus)^51^. A standard curve was generated for quantification of *C. pecorum* infection loads using a known concentration of *C. pecorum* genomic DNA diluted to 10^7^–10^1^ copies/µL, followed by a high-resolution melt (HRM) analysis. Samples with less than 10 copies/µL and with no or below threshold HRM were considered below the detectable limit of the assay and were reported as negative. Samples were reported as either positive or negative for the *C. pecorum* plasmid based on positive amplification and confirmation with an HRM curve at 79.0 °C + /−0.5 °C. Samples were run in duplicate, and positive and negative controls were included in all qPCR assays.

To evaluate the genetic diversity of infecting strains, two strain typing methods were employed. Availability and longitudinal data dictated which samples were analysed from MB and all HV samples were analysed. A 359 bp fragment of the V3/V4 regions of the *omp*A gene was amplified^9,10^ in 38 *C. pecorum*-positive samples from 28 koalas (14 from MB and 24 from HV). A *C. pecorum*-specific Multi-Locus Sequence Typing scheme, based on the seven housekeeping genes *gat*A, *opp*A_3, *hfl*X, *gid*A, *eno*A, *hem*N and *fum*C^27^, was applied to 69 *C. pecorum*-positive samples from 39 koalas (45 from MB and 24 from HV). The PCR products were electrophoresed on a 1.5% agarose gel, followed by visual confirmation under a UV transilluminator and amplicons were bidirectionally sequenced at Macrogen Inc (Korea). Sequence analyses were performed in Geneious Prime (2019.2.3) (https://www.geneious.com/), including Clustal X alignments and Bayesian phylogenetic analyses using Mr Bayes. *omp*A sequences were analysed by BLAST against the nr/nt database using megablast to determine their similarity to other publicly available *omp*A sequences, whereas MLST sequences were interrogated in ChlamydialesPubMLST as previously described^27^. The novel ST that was detected, ST 281, as well as the individual alleles, was deposited in the ChlamydialesPubMLST ref: https://www.ncbi.nlm.nih.gov/pubmed/30345391 (https://pubmlst.org/chlamydiales/).

### Sample analysis for major histocompatibility complex genes and koala retrovirus subtypes

Total DNA was extracted from 200 µL of blood serum clots using a QIAamp DNA mini kit (Qiagen), according to the manufacturer’s instructions. The DNA extracted from serum clots was used to amplify two Class I (*UA* and *UC*) and four Class II (*DAb*, *DBb*, *DCb*, *DMb*) MHC genes based on published primer sets^17,31^, as well as the receptor binding domain of the koala retrovirus *env* gene^46^ with PCR. The PCR products were electrophoresed on a 1.5% agarose gel, followed by visual confirmation under a UV transilluminator and amplicons were deep sequenced at the Ramaciotti Centre for Genomics (Australia). Forward and reverse amplicons were filtered, trimmed with cutadapt^52^ and merged with FLASH^53^. KoRV OTUs were determined with QIIME using UCLUST and USEARCH^54^ and BLAST searched against a known *env* gene library to allocate provirus subtypes^46^. Merged MHC sequences were aligned with Clustal X^55^ and trimmed in GeneDoc^56^ before being BLAST searched against a known MHC gene library to allocate alleles. Novel MHC gene sequences were translated to proteins to establish whether a new allele had been characterised, and novel MHC alleles were deposited with accession numbers MT321017-MT321064. MHC haplotype clusters were analysed with a Gower clustering analysis^57^ in RStudio 3.5.1 (2018.07.02) (https://www.R-project.org/) and compared to capture locations plotted on Google Earth. Neighbour-joining phylogenetic trees were generated in Geneious Prime (2019.02.03) (https://www.geneious.com/).

### Regulatory approvals

Koala management programs were conducted under approvals issued by the Queensland Department of Agriculture and Fisheries (approvals CA 2012/03/597, CA 2013/09/719, CA 2014/06/777, CA 2015/03/852, and CA 2016/03/950), and work with koalas was authorised by scientific purposes permits issued by the Queensland Department of Environment and Heritage Protection (approvals WISP 11525212, WISP 16125415, WISP 13661313, WITK 14173714, WISP 17273716 and WA 0008304). Swab samples were analysed under approval numbers AN/A/13/80 and AN/E/19/33 issued by the University of the Sunshine Coast Animal Ethics Committee. All experiments were performed in accordance with the relevant guidelines and regulations.

### Statistical analyses

Statistical analyses were performed using the Kruskal-Walis test, Mann–Whitney U test, two-tailed Fisher’s Exact test, chi-square test and independent t-test as appropriate. Data were not normally distributed for any parameter except KoRV-A diversity, and some analyses were limited by small sample size. All statistical analyses were performed with the IBM SPSS Statistics software package (version 26, https://www.ibm.com/products/spss-statistics), and the statistical significance of all tests was concluded at *p-values* of < 0.05.

## Supplementary information

Supplementary file1

Supplementary file2

Supplementary file3

## References

[1] Beyer HL (2018). Management of multiple threats achieves meaningful koala conservation outcomes. J. Appl. Ecol..

[2] Polkinghorne A, Hanger J, Timms P (2013). Recent advances in understanding the biology, epidemiology and control of chlamydial infections in koalas. Vet. Microbiol..

[3] Jackson M, White N, Giffard P, Timms P (1999). Epizootiology of *Chlamydia* infections in two free-range koala populations. Vet. Microbiol..

[4] 4Hanger, J. *et al.* Moreton Bay Rail Koala Management Program: Final Technical Report for Queensland Department of Transport and Main Roads., 1–351 (Toorbul, Queensland, 2017).

[5] Burach F (2014). Chlamydiaceae and Chlamydia-like organisms in the koala (*Phascolarctos cinereus*)–organ distribution and histopathological findings. Vet. Microbiol..

[6] Pagliarani S (2020). Chlamydia pecorum infection in the reproductive tract of female koalas (*Phascolarctos cinereus*). J. Comp. Pathol..

[7] Palmieri C (2018). *Chlamydia pecorum* infection in the male reproductive system of koalas (*Phascolarctos cinereus*). Vet. Pathol..

[8] Rhodes JR (2011). Using integrated population modelling to quantify the implications of multiple threatening processes for a rapidly declining population. Biol. Cons..

[9] Robbins A, Hanger J, Jelocnik M, Quigley BL, Timms P (2019). Longitudinal study of wild koalas (*Phascolarctos cinereus*) reveals chlamydial disease progression in two thirds of infected animals. Sci. Rep..

[10] Nyari S (2017). Epidemiology of chlamydial infection and disease in a free-ranging koala (*Phascolarctos cinereus*) population. PLoS ONE.

[11] Legione AR (2016). Identification of unusual *Chlamydia pecorum* genotypes in Victorian koalas (*Phascolarctos cinereus*) and clinical variables associated with infection. J. Med. Microbiol..

[12] Fernandez CM (2019). Differences in the genetic diversity of Chlamydia pecorum between neighbouring sub-populations of koalas (*Phascolarctos cinereus*): a potential issue for wildlife corridor construction in population management. Vet. Microbiol..

[13] Quigley BL, Carver S, Hanger J, Vidgen ME, Timms P (2018). The relative contribution of causal factors in the transition from infection to clinical chlamydial disease. Sci. Rep..

[14] Wan C (2011). Using quantitative polymerase chain reaction to correlate *Chlamydia pecorum* infectious load with ocular, urinary and reproductive tract disease in the koala (*Phascolarctos cinereus*). Aust. Vet. J..

[15] Waugh CA (2017). Infection with koala retrovirus subgroup B (KoRV-B), but not KoRV-A, is associated with chlamydial disease in free-ranging koalas (*Phascolarctos cinereus*). Sci. Rep..

[16] Legione AR (2017). Koala retrovirus genotyping analyses reveal a low prevalence of KoRV-A in Victorian koalas and an association with clinical disease. J. Med. Microbiol..

[17] Lau Q, Griffith JE, Higgins DP (2014). Identification of MHCII variants associated with chlamydial disease in the koala (*Phascolarctos cinereus*). PeerJ.

[18] Patterson JL (2015). The prevalence and clinical significance of *Chlamydia* infection in island and mainland populations of Victorian koalas (*Phascolarctos cinereus*). J. Wildl. Dis..

[19] Fabijan J (2019). Chlamydia pecorum prevalence in South Australian koala (*Phascolarctos cinereus*) populations: identification and modelling of a population free from infection. Sci. Rep..

[20] Bryant LM, Krosch MN (2016). Lines in the land: a review of evidence for eastern Australia's major biogeographical barriers to closed forest taxa. Biol. J. Lin. Soc..

[21] Lee KE (2013). Anthropogenic changes to the landscape resulted in colonization of koalas in north-east New South Wales, Australia. Austral. Ecol..

[22] Kjeldsen SR (2018). Genomic comparisons reveal biogeographic and anthropogenic impacts in the koala (*Phascolarctos cinereus*): a dietary-specialist species distributed across heterogeneous environments. Heredity.

[23] Johnson RN (2018). Adaptation and conservation insights from the koala genome. Nat. Genet..

[24] Neaves LE (2016). Phylogeography of the Koala, (*Phascolarctos cinereus*), and harmonising data to inform conservation. PLoS ONE.

[25] Koroleva E (2017). Chlamydial type III secretion system needle protein induces protective immunity against chlamydia muridarum intravaginal infection. Biomed. Res. Int..

[26] Quigley BL, Ong VA, Hanger J, Timms P (2018). Molecular dynamics and mode of transmission of Koala retrovirus as it invades and spreads through a Wild Queensland Koala population. J. Virol..

[27] Jelocnik M., P. A., Pannekoek Y. In *Chlamydia trachomatis. Methods in Molecular Biology.* Vol. 2042 (Humana, 2019).

[28] Higgins DP, Hemsley S, Canfield PJ (2005). Association of uterine and salpingeal fibrosis with chlamydial hsp60 and hsp10 antigen-specific antibodies in *Chlamydia*-infected koalas. Clin. Diagn. Lab. Immunol..

[29] Poston TB (2019). Cervical cytokines associated with chlamydia trachomatis susceptibility and protection. J. Infect. Dis..

[30] Murthy AK, Li W, Ramsey KH (2018). Immunopathogenesis of chlamydial infections. Curr. Top. Microbiol. Immunol..

[31] Cheng YY (2018). Characterisation of MHC class I genes in the koala. Immunogenetics.

[32] Wedrowicz F, Mosse J, Wright W, Hogan FE (2018). Using non-invasive sampling methods to determine the prevalence and distribution of Chlamydia pecorum and koala retrovirus in a remnant koala population with conservation importance. Wildl. Res..

[33] Jelocnik M, Frentiu FD, Timms P, Polkinghorne A (2013). Multilocus sequence analysis provides insights into molecular epidemiology of *Chlamydia pecorum* infections in Australian sheep, cattle, and koalas. J. Clin. Microbiol..

[34] Mohamad K (2008). Preliminary phylogenetic identification of virulent *Chlamydophila pecorum* strains. Infect. Genet. Evol..

[35] Higgins DP, Beninati T, Meek M, Irish J, Griffith JE (2012). Within-population diversity of koala *Chlamydophila pecorum* at *omp*A VD1-VD3 and the *ORF663* hypothetical gene. Vet. Microbiol..

[36] Mohamad KY (2014). Host adaptation of *Chlamydia pecorum* towards low virulence evident in co-evolution of the *ompA*, *incA*, and *ORF663* Loci. PLoS ONE.

[37] Kaltenboeck B, Heinen E, Schneider R, Wittenbrink M, Schmeer N (2009). OmpA and antigenic diversity of bovine *Chlamydophila pecorum* strains. Vet. Microbiol..

[38] Bachmann NL (2015). Culture-independent genome sequencing of clinical samples reveals an unexpected heterogeneity of infections by *Chlamydia pecorum*. J. Clin. Microbiol..

[39] Bachmann NL, Polkinghorne A, Timms P (2014). *Chlamydia* genomics: providing novel insights into chlamydial biology. Trends Microbiol..

[40] Marsh J, Kollipara A, Timms P, Polkinghorne A (2011). Novel molecular markers of *Chlamydia pecorum* genetic diversity in the koala (*Phascolarctos cinereus*). BMC Microbiol..

[41] Tarlinton R, Meers J, Young P (2006). Retroviral invasion of the koala genome. Nature.

[42] Avila-Arcos M (2013). One hundred twenty years of koala retrovirus evolution determined from museum skins. Mol. Biol. Evol..

[43] Tarlinton, R. E. *et al.* Differential and defective expression of Koala Retrovirus reveal complexity of host and virus evolution. *bioRxiv*, 211466. 10.1101/211466 (2017).

[44] Chappell K (2017). Phylogenetic diversity of koala retrovirus within a Wild Koala population. J. Virol..

[45] Sarker N (2020). Koala retrovirus viral load and disease burden in distinct northern and southern koala populations. Sci. Rep..

[46] Quigley, B. *et al.* Changes in endogenous and exogenous koala retrovirus subtype expression over time reflect koala health outcomes. *J. Virol.* **93**, undefined-undefined, 10.1128/JVI.00849-19 (2019). 10.1128/JVI.00849-19 PMC6714790 31243137

[47] Seabrook L (2011). Drought-driven change in wildlife distribution and numbers: a case study of koalas in south west Queensland. Wildl. Res..

[48] Gordon G, Brown A, Pulsford T (1988). A koala (*Phascolarctos cinereus* Goldfuss) population crash during drought and heat-wave conditions in southwestern Queensland. Aust. J. Ecol..

[49] Robbins A, Loader J, Timms P, Hanger J (2018). Optimising the short and long-term clinical outcomes for koalas (*Phascolarctos cinereus*) during treatment for chlamydial infection and disease. PLoS ONE.

[50] Jelocnik M (2017). Development and evaluation of rapid novel isothermal amplification assays for important veterinary pathogens: *Chlamydia psittaci* and *Chlamydia pecorum*. PeerJ.

[51] Phillips S (2018). *Chlamydia pecorum* gastrointestinal tract infection associations with urogenital tract infections in the koala (*Phascolarctos cinereus*). PLoS ONE.

[52] Martin M (2011). Cutadapt removes adapter sequences from high-throughput sequencing reads. EMBnet. J..

[53] Magoc T, Salzberg SL (2011). FLASH: fast length adjustment of short reads to improve genome assemblies. Bioinformatics.

[54] Edgar RC (2010). Search and clustering orders of magnitude faster than BLAST. Bioinformatics.

[55] Larkin MA (2007). Clustal W and Clustal X version 2.0. Bioinformatics.

[56] Nicholas, K., Nicholas, H. G., Nicholas, K.B., Nicholas, H.B., Deerfield, D.W., Nicholas, H.B.J., Nicholas, H.J., Nicholas, K.R., Nicholas, H.B.J., Nicholas, A., Deerfield, D.W., Nicholas, H., Gauch, H. . GeneDoc: a tool for editing and annotating multiple sequence alignments. *Embnet.news* (1997).

[57] Gower JC (1967). A comparison of some methods of cluster analysis. Biometrics.

