# Supplementary material for: Koala immunogenetics and chlamydial strain type are more directly involved in chlamydial disease progression in koalas from two south east Queensland koala populations than koala retrovirus subtypes
Source: Sci Rep. 2020 Sep 14;10:15013. doi: 10.1038/s41598-020-72050-2 (PMC7490398; doi:10.1038/s41598-020-72050-2)
Supplement: Supplementary file 2 — Supplementary file2 [file 41598_2020_72050_MOESM2_ESM.pdf]

Koala immunogenetics and chlamydial strain type are more directly involved in chlamydial disease progression in koalas from two south east Queensland koala populations than koala retrovirus subtypes

Amy Robbins[1,2], Jonathan Hanger[2], Martina Jelocnik[1], Bonnie L. Quigley[1], Peter Timms[1]*

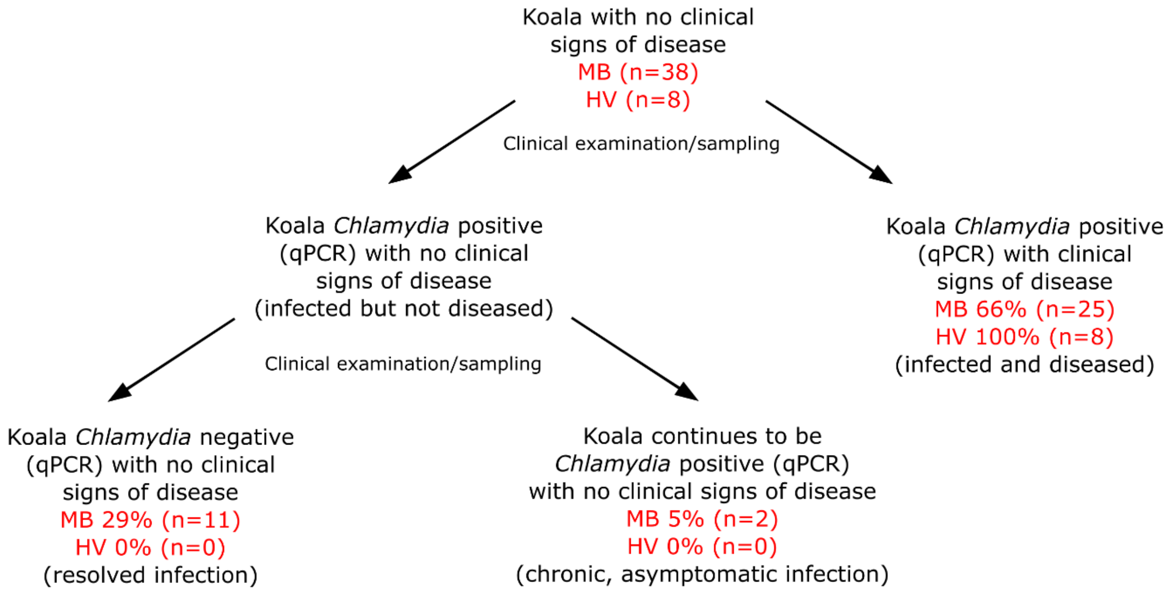

Supplementary Figure S1: A comparison of the outcomes of new chlamydial infections at the Moreton Bay site (MB) and the Old Hidden Vale site (HV)

a

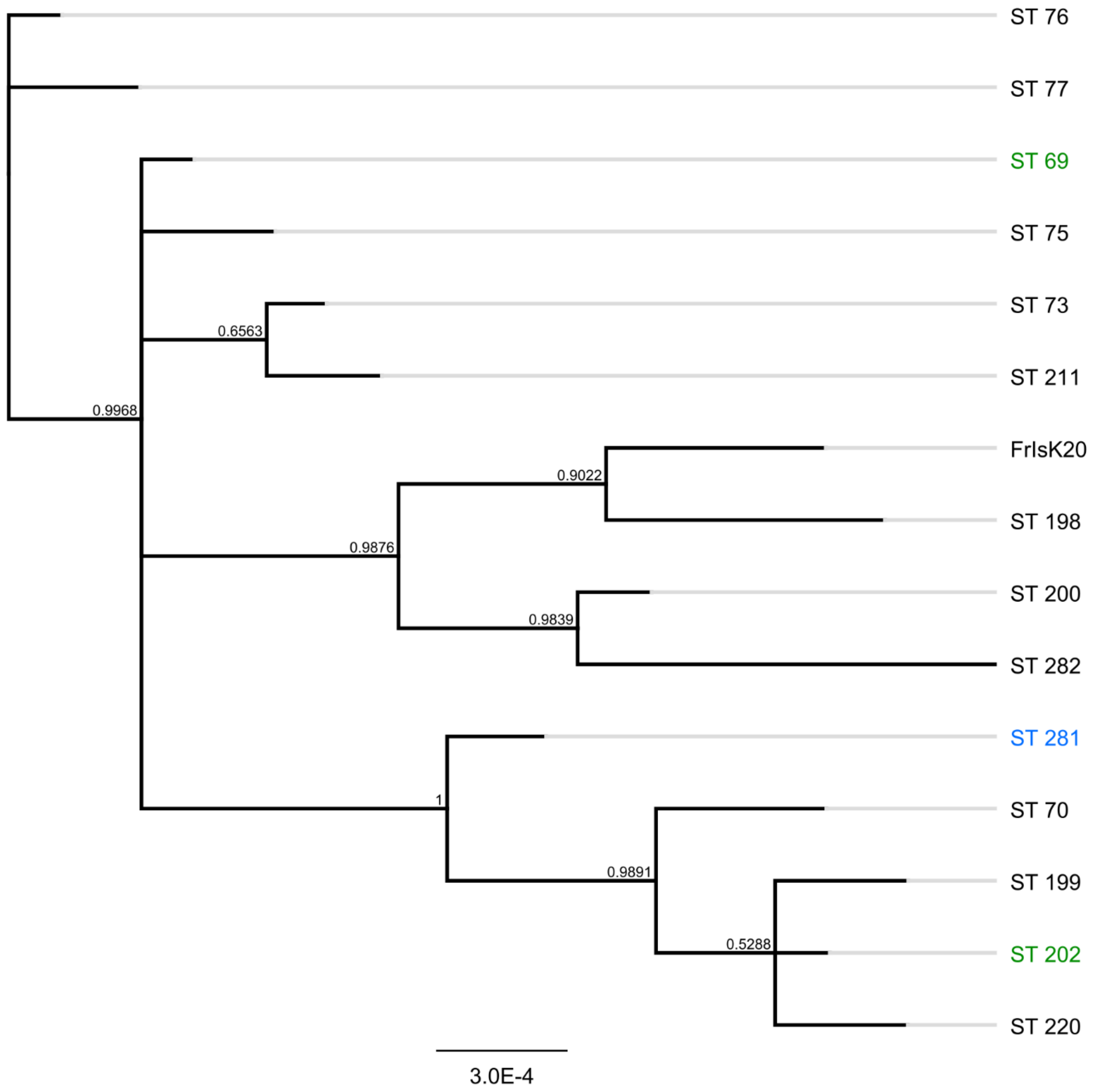

ST 76
ST 77
ST 69
ST 75
ST 73
ST 211
FrIsK20
ST 198
ST 200
ST 282
ST 281
ST 70
ST 199
ST 202
ST 220
0.6563
0.9968
0.9022
0.9876
0.9839
1
0.9891
0.5288
3.0E-4

b

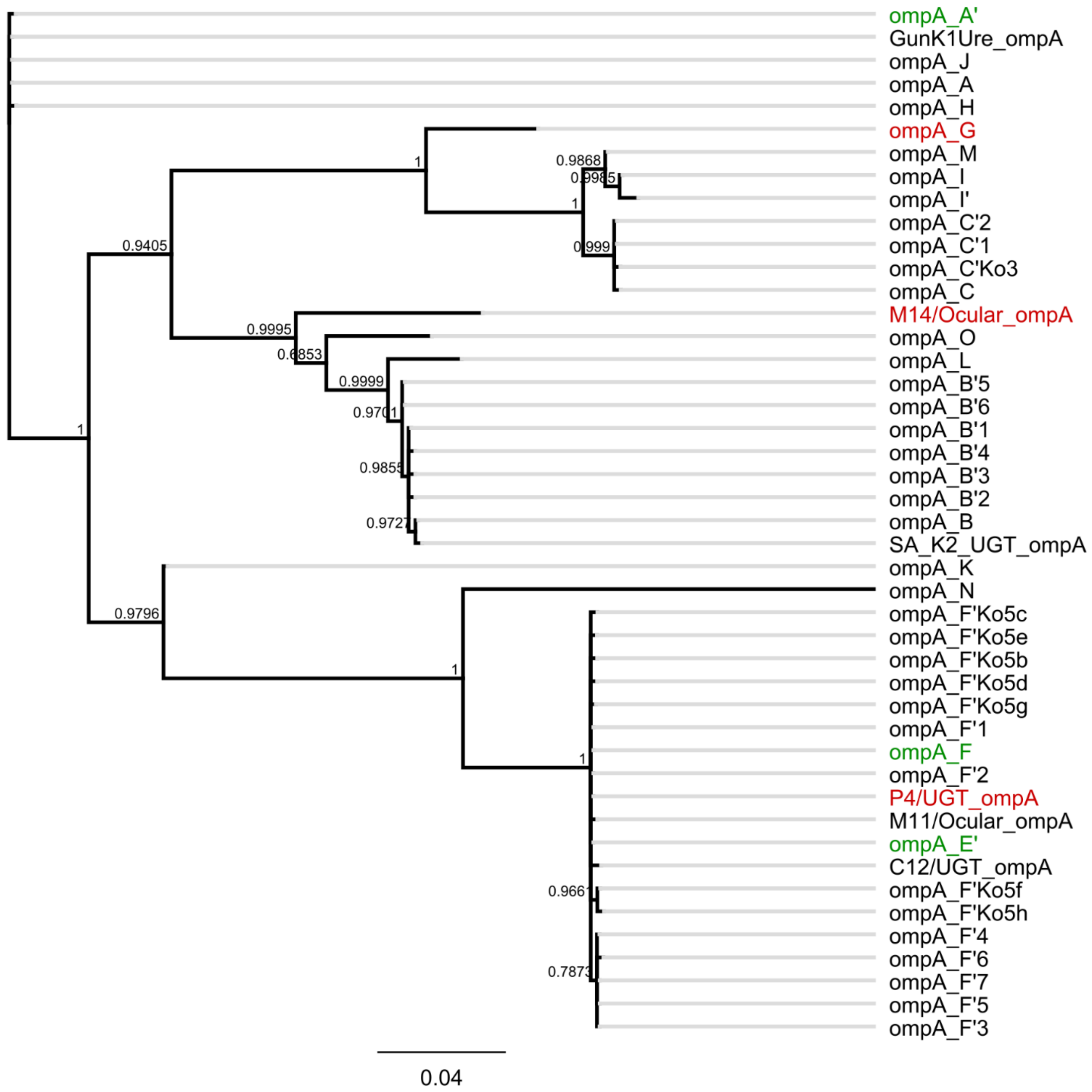

Supplementary Figure S2: Mid-point rooted Bayesian phylogenetic trees of a. Multi-Locus Sequence Typing scheme sequence types (ST) and b. *omp*A genotypes detected in koalas, indicating the sequences that were detected in this study (red denotes Moreton Bay site (MB) only, blue denotes Old Hidden Vale site (HV) only, green denotes both study sites)

a

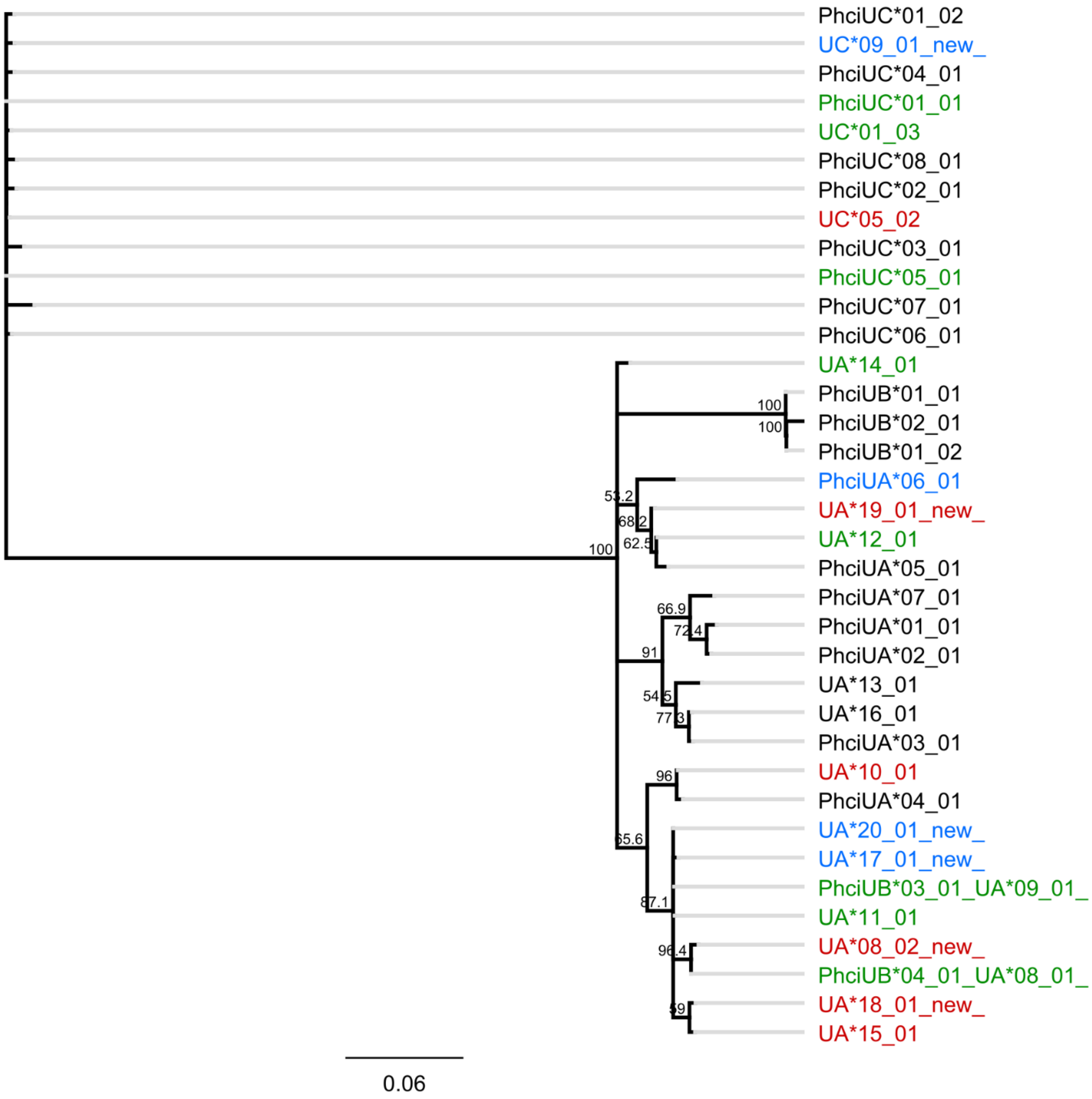
PhciUC*01_02
UC*09_01_new_
PhciUC*04_01
PhciUC*01_01
UC*01_03
PhciUC*08_01
PhciUC*02_01
UC*05_02
PhciUC*03_01
PhciUC*05_01
PhciUC*07_01
PhciUC*06_01
UA*14_01
PhciUB*01_01
PhciUB*02_01
PhciUB*01_02
PhciUA*06_01
UA*19_01_new_
UA*12_01
PhciUA*05_01
PhciUA*07_01
PhciUA*01_01
PhciUA*02_01
UA*13_01
UA*16_01
PhciUA*03_01
UA*10_01
PhciUA*04_01
UA*20_01_new_
UA*17_01_new_
PhciUB*03_01_UA*09_01_
UA*11_01
UA*08_02_new_
PhciUB*04_01_UA*08_01_
UA*18_01_new_
UA*15_01
100
100
100
68.2
62.5
66.9
72.4
91
77.3
96
87.1
96.4
0.06

b

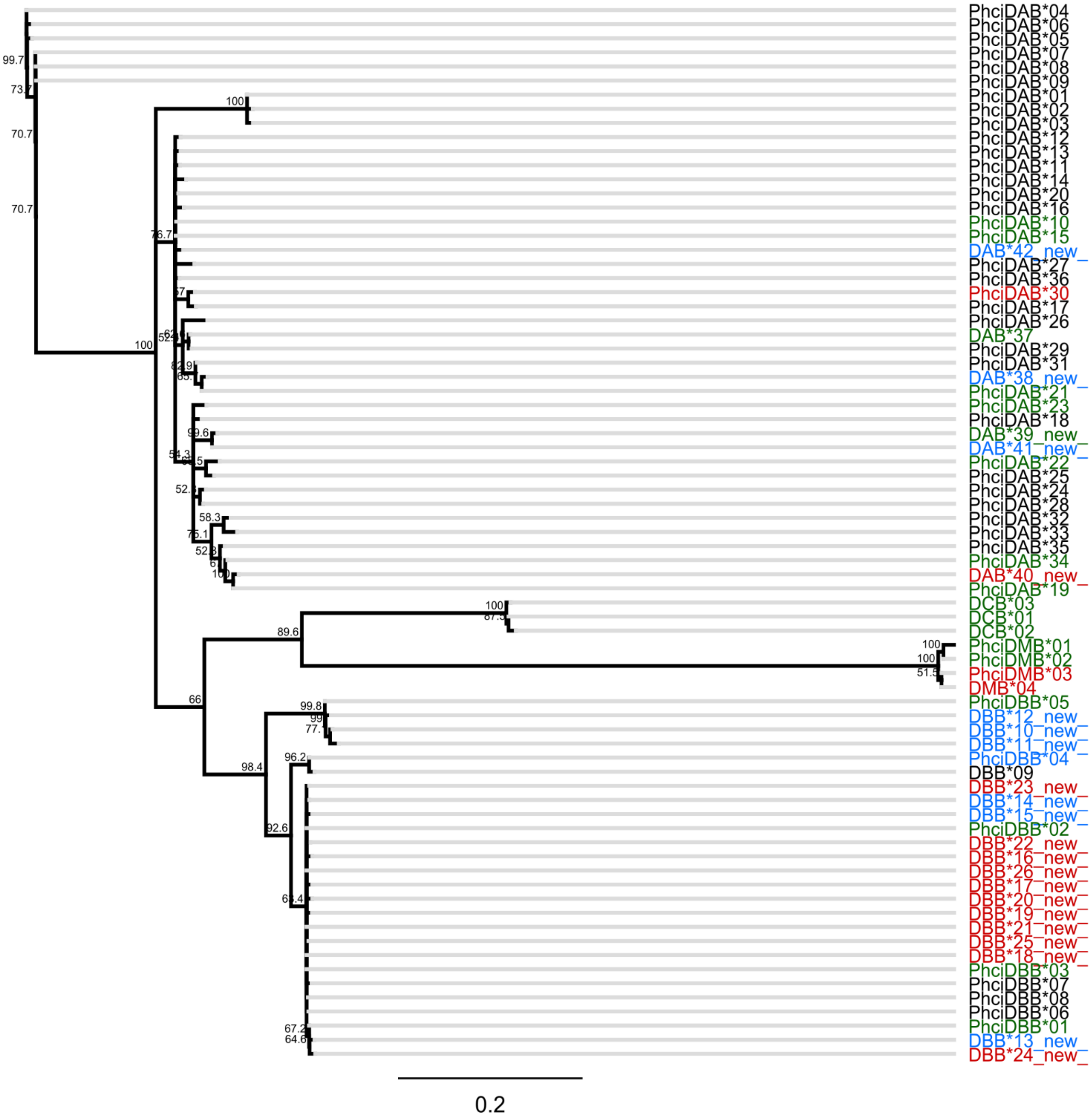

Supplementary Figure S3: Mid-point rooted Neighbour-joining phylogenetic trees of a. Class I and b. Class II major histocompatibility complex alleles indicating novel alleles identified in this study and the distribution of alleles between the Moreton Bay site (MB) and the Old Hidden Vale site (HV) (red denotes Moreton Bay site only, blue denotes Old Hidden Vale site only, green denotes both study sites)

Supplementary Table S1: The prevalence of major histocompatibility complex (MHC) alleles at the Moreton Bay site (MB) and the Old Hidden Vale site (HV) that were significantly different between the study sites

MHC allele	Moreton Bay site (MB)	Old Hidden Vale site (HV)	Fisher's exact test significance
UA 08:01	52% (31/60)	90% (18/20)	*p*=0.003
UA 10:01	45% (27/60)	0% (0/20)	*p*<0.001
UA 11:01	10% (6/60)	45% (9/20)	*p*=0.001
UA 14:01	32% (19/60)	5% (1/20)	*p*=0.017
UA 17:01	0% (0/60)	15% (3/20)	*p*=0.014
UC 01:01	100% (60/60)	80% (16/20)	*p*=0.003
UC 01:03	8% (5/60)	30% (6/20)	*p*=0.024
UC 05:02	30% (18/60)	0% (0/20)	*p*=0.004
DAb 10	35% (21/60)	5% (1/20)	*p*=0.009
DAb 19	88% (53/60)	60% (12/20)	*p*=0.009
DAb 22	2% (1/60)	15% (3/20)	*p*=0.046
DAb 23	2% (1/60)	20% (4/20)	*p*=0.013
DAb 37	2% (1/60)	20% (4/20)	*p*=0.013
DBb 03	53% (32/60)	15% (3/20)	*p*=0.004
DBb 04	0% (0/60)	20% (4/20)	*p*=0.003
DMb 04	25% (15/60)	0% (0/20)	*p*=0.017

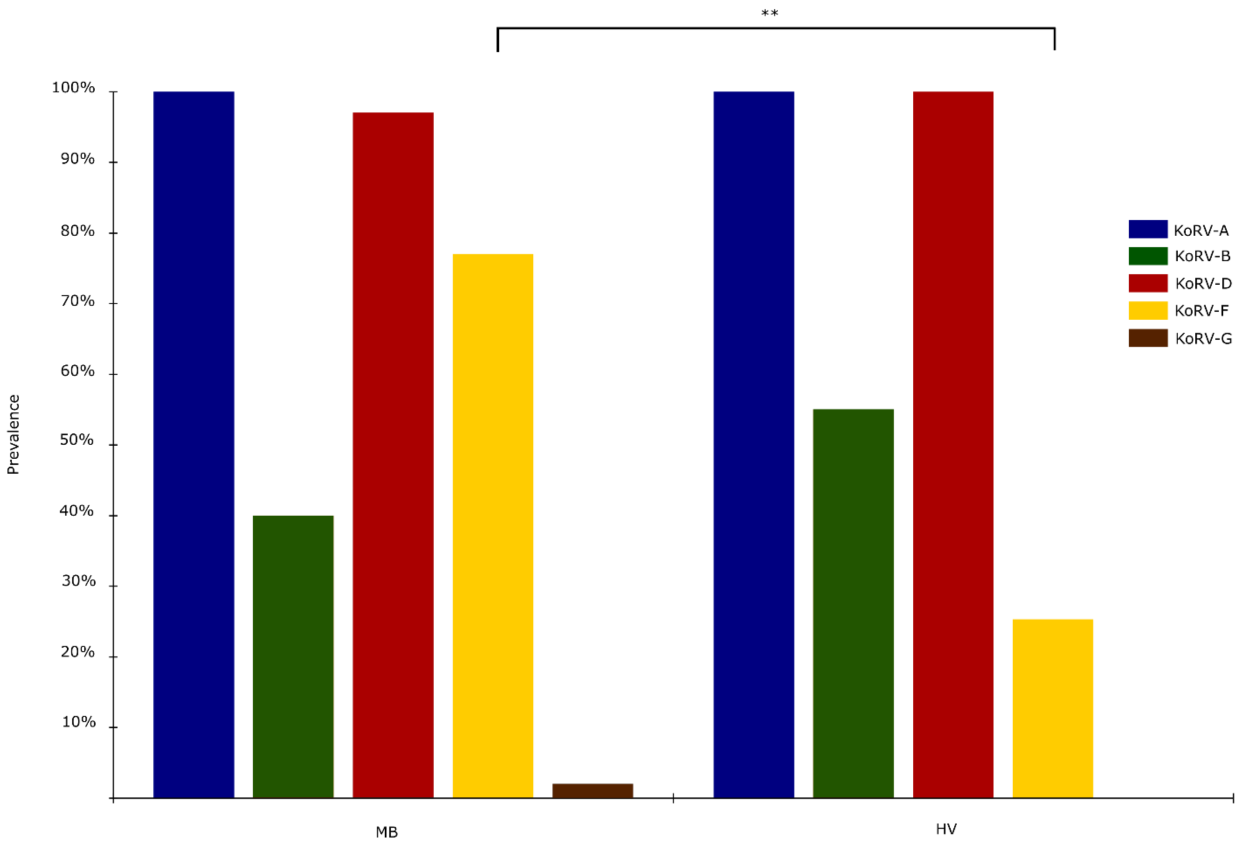

Supplementary Figure S4: A comparison of the prevalence of KoRV subtypes between the Moreton Bay site (MB) and the Old Hidden Vale site (HV), Fisher's exact test ***p*<0.001
