# Supplementary material for: Koala immunogenetics and chlamydial strain type are more directly involved in chlamydial disease progression in koalas from two south east Queensland koala populations than koala retrovirus subtypes
Source: Sci Rep. 2020 Sep 14;10:15013. doi: 10.1038/s41598-020-72050-2 (PMC7490398; doi:10.1038/s41598-020-72050-2)
Supplement: Supplementary file 3 — Supplementary file3 [file 41598_2020_72050_MOESM3_ESM.pdf]

# Koala immunogenetics and chlamydial strain type are more directly involved in chlamydial disease progression in koalas from two south east Queensland koala populations than koala retrovirus subtypes

Amy Robbins[1,2], Jonathan Hanger[2], Martina Jelocnik[1], Bonnie L. Quigley[1], Peter Timms[1]*

***Prior chlamydial exposure was not necessary for 'severe' chlamydial disease or female reproductive tract disease at the Old Hidden Vale site***

There were no significant differences between the study sites in the number of diseased koalas who developed 'severe' disease (defined as disease that warranted euthanasia on welfare grounds due to a poor prognosis, resulted in the death of the koala, or would have without veterinary intervention) with 21% of diseased koalas (3/14) at HV and 10% of diseased koalas (16/158) at MB [9] affected (Fisher's exact test *p*=0.189). All MB and HV koalas with 'severe' chlamydial disease had either cystitis or cystitis and reproductive or ocular disease (multifocal disease). There were no significant differences in the median age (HV Mann-Whitney U=6, *p*=0.383), sex (HV Fisher's exact test *p*=1.000) or infection load (HV Mann-Whitney U=2, *p*=0.067) between diseased koalas and those affected by 'severe' disease at either study site [9]. In contrast to MB, however, where 100% of koalas (5/5) with 'severe' disease had previously received antibiotic treatment for chlamydial infection or disease [9], none of the koalas at HV with 'severe' disease (0%, 0/3) had previously received treatment.

The number of diseased female koalas diagnosed with reproductive tract disease was not significantly different between the study sites, with 86% of the diseased female koalas (6/7) at HV and 63% of the diseased female koalas (62/98) at MB [9] affected (Fisher's exact test, *p*=0.417). Prolonged or repeated exposure to chlamydial antigens was not necessary for female reproductive tract disease at HV, and 50% of the diseased female koalas diagnosed with reproductive tract disease (3/6) at HV were under 2.5 years of age. This was not significantly different to MB (Fisher's exact test *p*=0.683), where 24% of the diseased female koalas diagnosed with reproductive tract disease (15/62) acquired their infection shortly after sexual maturity [9]. In contrast to MB, however, where only 53% of the female koalas with clinically detectable reproductive tract disease (16/30) had a detectable infection at their urogenital tract site [9],

100% of the female koalas with clinically detectable reproductive tract disease (6/6) at HV were infected (Fisher's exact test $p$=0.063).

***At both study sites, antimicrobial treatment provided only short-term 'protection'***

Successful antimicrobial treatment at HV provided only short-term (less than 197 days) 'protection' against reinfection and subsequent disease progression in susceptible individuals (n=3). Similarly, successful antimicrobial treatment at MB provided only short-term 'protection' against reinfection (less than 116 days) and subsequent disease progression (less than 131 days) [9]. Following one HV koala's release after successful antimicrobial treatment for chlamydial infection and disease, she re-presented (approx. 6 months later) with a newly acquired chlamydial infection and episode of disease. Although an identical *omp*A genotype was detected in each infection, the STs differed (Fig. 2).

***A second novel Multi-Locus Sequence Typing sequence type may have been present at the Moreton Bay site***

Our data suggest that a second novel ST was present at MB. The gene sequence for *gid*A allele 38 was detected in a single ocular site sample from one koala (2%, 1/45) and this allele has not previously been reported in koalas (Supplementary Fig. S5). Unfortunately, four of the seven other housekeeping genes in the MLST scheme could not be resolved in this sample, so a ST could not be allocated. Interestingly, this was also the only sample that tested negative for the *C. pecorum* plasmid.

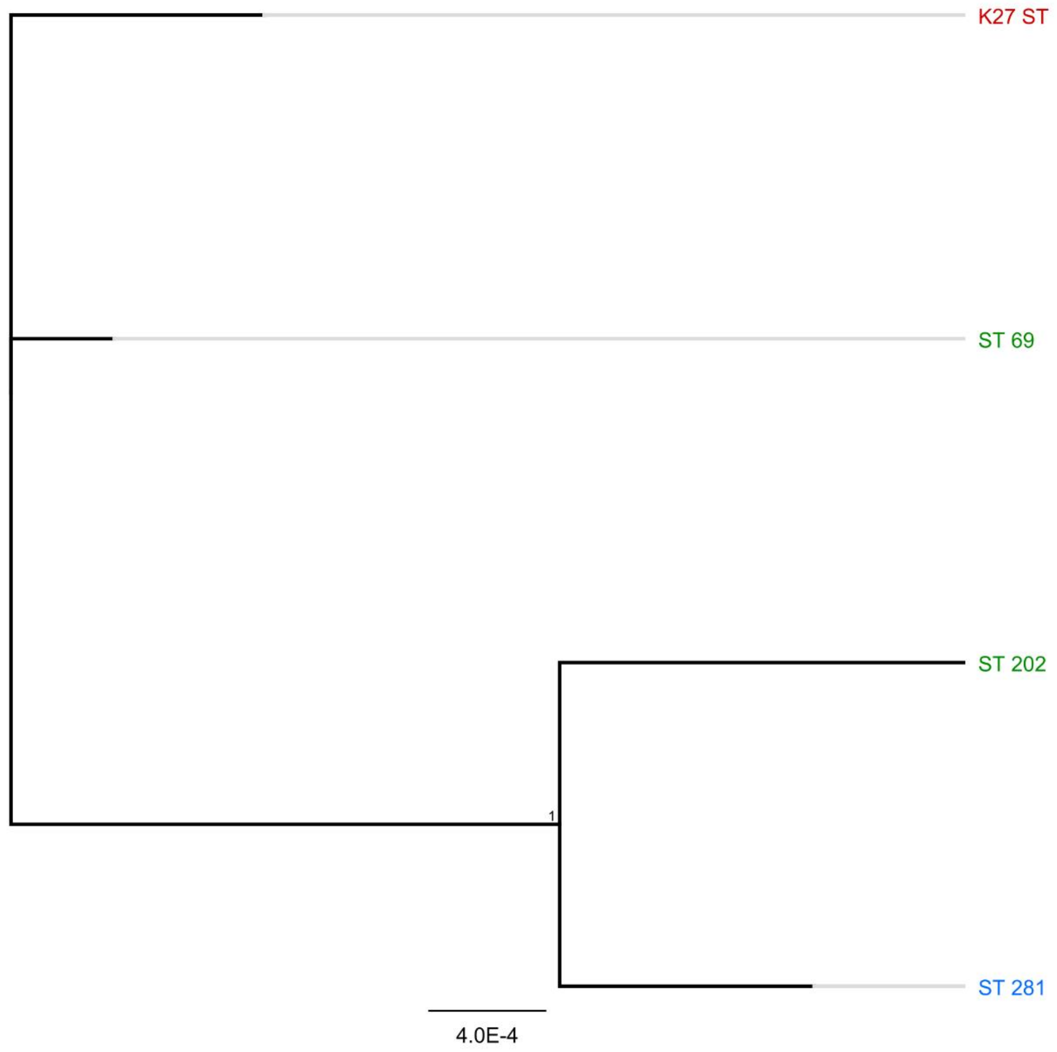


Supplementary Figure S5: Mid-point rooted Bayesian phylogenetic tree of an alignment of three concatenated Multi-Locus Sequence Typing genes, *gat*A, *gid*A and *eno*A, detected in this study (red denotes Moreton Bay site only (MB), blue denotes Old Hidden Vale site only (HV) and green denotes both study sites)

***There were significant differences in the diversity of some KoRV subtypes between the study sites***

Overall, 314 OTUs were identified at both study sites, 250 OTUs at MB and 150 OTUs at HV. KoRV-A was significantly more diverse at MB (13 KoRV-A OTUs per koala, range 7-19 KoRV-A OTUs per koala) compared to HV (10 KoRV-A OTUs per koala, range 7-13 KoRV-A OTUs per koala) (independent t-test $t(-4.921)$, $p<0.001$) (Supplementary Fig. S6). KoRV-F was also significantly more diverse at MB (2 KoRV-F OTUs per koala, range 0-18 KoRV-F OTUs per koala) compared to HV (0 KoRV-F OTUs per koala, range 0-2 KoRV-F

OTUs per koala) (Mann-Whitney U=313.5, $p<0.001$). There were no significant differences in the diversity of KoRV-B, -D or -G between the study sites.

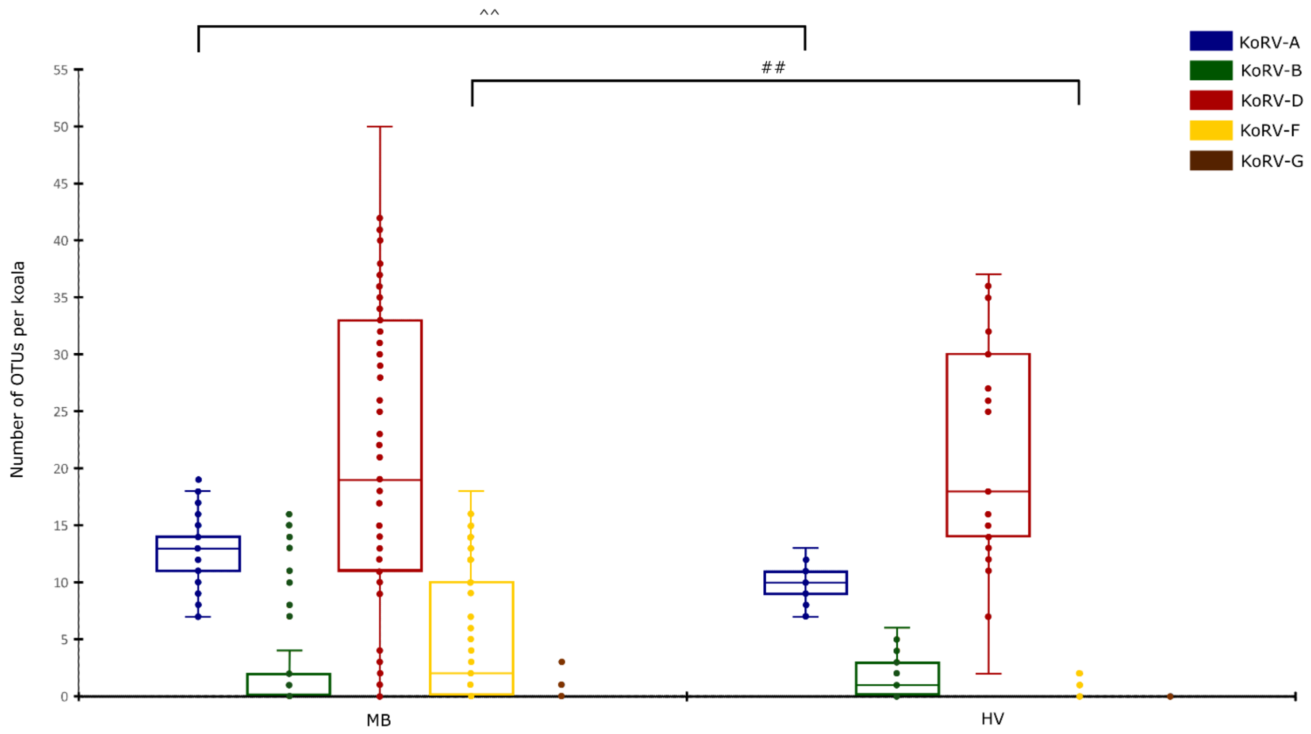


Supplementary Figure S6: A comparison of the diversity of koala retrovirus (KoRV) subtypes, as determined by the number of operational taxonomic units (OTUs) per koala, between the Moreton Bay site (MB) and the Old Hidden Vale site (HV), independent t-test ^^$p<0.001$, Mann-Whitney U test ##$p<0.001$

## *KoRV profiles were generally stable over time*

Overall, longitudinal samples were collected from 12 koalas (9 MB and 3 HV koalas) for KoRV profile analyses. There were only minor changes in the KoRV profiles of eleven of these koalas, with the acquisition or loss of no more than 10 OTUs of any KoRV subtype over time. The remaining koala, a male who was translocated to a new area (due to management reasons), had a markedly different KoRV profile before and after translocation. He acquired five new KoRV-A OTUs and 23 new KoRV-D OTUs and lost 1 KoRV-A OTU. Subsequently, the proportional abundance of KoRV-A declined from 100% (270,249/270,254) to 97.85% (142,176/145,293) and the proportional abundance of KoRV-D increased from less than 0.01% (4/270,254) to 2.14% (3,115/145,293).
